# Cyclin E1 and Rb modulation as common events at time of resistance to palbociclib in hormone receptor-positive breast cancer

**DOI:** 10.1038/s41523-018-0092-4

**Published:** 2018-11-28

**Authors:** Cristina Guarducci, Martina Bonechi, Matteo Benelli, Chiara Biagioni, Giulia Boccalini, Dario Romagnoli, Roberto Verardo, Rachel Schiff, C. Kent Osborne, Carmine De Angelis, Angelo Di Leo, Luca Malorni, Ilenia Migliaccio

**Affiliations:** 1 “Sandro Pitigliani” Translational Research Unit, Hospital of Prato, Prato, Italy; 2 Bioinformatics Unit, Hospital of Prato, Prato, Italy; 3 “Sandro Pitigliani” Medical Oncology Department, Hospital of Prato, Prato, Italy; 4 Laboratorio Nazionale CIB (LNCIB), Area Science Park, Functional Genomics & Bioinformatics Units, Trieste, Italy; 5 Lester and Sue Smith Breast Center, Baylor College of Medicine, Houston, TX USA; 6 Dan L Duncan Comprehensive Cancer Center, Baylor College of Medicine, Houston, TX USA; 7 Department of Molecular and Cellular Biology, Baylor College of Medicine, Houston, TX USA; 8 Department of Medicine, Baylor College of Medicine, Houston, TX USA

## Abstract

CDK4/6 inhibitors represent a new treatment standard for hormone receptor-positive (HR+), HER2-negative advanced breast cancer (BC) patients. Although efficacious, resistance to these agents is universal. Here, we profiled a large panel of HR+ BC cell lines with conditioned resistance to the CDK4/6 inhibitor palbociclib, and analyzed cell cycle-related markers by gene expression profiles (GEP) and western blot (WB). GEP showed high molecular heterogeneity among the models, with E2F targets being significantly enriched both during treatment and at the time of resistance. By both WB and GEP, a common molecular feature at the time of palbociclib resistance was the concomitant overexpression of cyclin E1 and down-regulation of Rb. *CCNE1* was the only significantly up-regulated gene among E2F targets at resistance with *CCNE1* genomic amplification being observed in two resistant models. Rb was downregulated in all resistant models; a reduction of *RB1* copy number was observed in three resistant cell lines. In silico analyses showed that *CCNE1/RB1* ratio correlated with palbociclib IC50 in different datasets of both breast and non-breast cancer cell lines, performing better than *CCNE1* or *RB1* taken separately. Finally, the *CCNE1/RB1* ratio was shown to be an adverse prognostic factor in patients with ER+ BC and to be able to discriminate palbociclib-sensitive versus resistant among patients enrolled in the NeoPalAna trial, a neoadjuvant trial testing palbociclib, performing better than *CCNE1* or *RB1* alone. Our data suggest that the *CCNE1/RB1* ratio may be a viable biomarker of palbociclib resistance, warranting further clinical validation.

## Biomarkers: Prognostic indicator for resistance to cell cycle–targeted drug

The expression levels of two genes involved in controlling the cell cycle offers a promising predictive indicator of whether women with hormone receptor–positive breast cancer are likely to develop resistance to the drug palbociclib. A team led by Luca Malorni and Ilenia Migliaccio from the Hospital of Prato, Italy, analyzed gene and protein profiles in a panel of breast cancer cell lines with differential sensitivity to palbociclib. The researchers found that, compared to palbociclib-sensitive cell lines, those that were drug-resistant tended to have elevated expression of one cell-cycle–related gene, called *CCNE1*, and reduced expression of another, called *Rb*. The ratio of *CCNE1:Rb* expression proved to be an accurate prognostic factor among patients enrolled in a clinical trial of palbociclib, performing better than either *CCNE1* or *Rb* levels on their own.

## Introduction

Palbociclib is a highly specific, orally active CDK4/6 inhibitor currently approved for the treatment of hormone receptor-positive, HER2-negative (HR+/HER2neg) advanced breast cancer (BC) in combination with the endocrine agents letrozole or fulvestrant.^1–3^ Given the efficacy and the tolerability shown by CDK4/6 inhibitors, utilization of these drugs in clinical practice is common in patients with HR+/HER2neg advanced BC. However, acquired resistance to these agents is universal, and results from clinical trials indicate that approximately 10 to 15% of patients may exhibit de novo resistance.^1–4^

CDK4 and CDK6 are kinases activated by binding to D-type cyclins, bearing a crucial role in cell proliferation through the regulation of cell cycle entry.^5^ The primary target of CDK4/6 action is the retinoblastoma susceptibility gene product (Rb) and other Rb family members (such as p107 and p130).^6^ Phosphorylation of Rb (pRb) by active cyclin-CDK complexes leads to release of transcription factors of the E2F family, and transcription of genes required for S-phase entry.^6^ Constraint upon CDK activity and G1 progression is provided by the universal CDK inhibitors of the Cip-Kip family, including p21 and p27, and the specific CDK4/6 inhibitors of the Ink family typified by p16.^7^

Preclinical data have already shown that deregulation of cell cycle genes and proteins is associated with resistance to palbociclib, including overexpression of cyclin E1 and loss of Rb.^8–10^ However, these events have been observed only in a limited number of in vitro and in vivo models, and these data have not been fully validated in the clinical setting. Therefore, the aim of our study is to further investigate if common cell cycle alterations, especially in the Rb/E2F pathway, could be observed in palbociclib-resistant models using a large panel of HR+ BC cell lines.

## Results

### Functional characterization of palbociclib-resistant (PDR) and palbociclib-sensitive (PDS) BC cell lines

We challenged eight BC cell lines with palbociclib including T47D, ZR75-1, MCF7, MCF7 Estrogen Deprivation Resistant (MCF7 EDR), MCF7 Tamoxifen Resistant (MCF7 TamR), CAMA1, MDA MB 361, and BT474. We developed PDR derivatives for all BC cell lines with the exception of CAMA1, which failed to restore growth with 350 nM palbociclib over two separate attempts. The time to develop resistance for all other models ranged from 10 to 27 weeks.

**Fig. 1:**
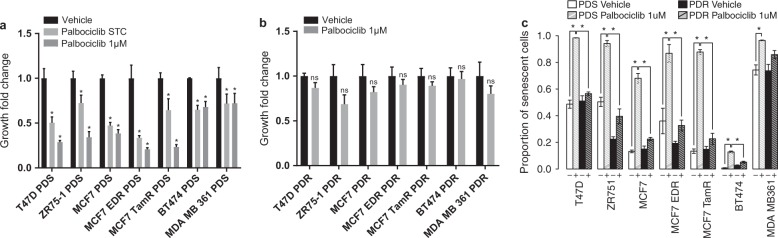
Functional characterization of PDR and PDS BC cell lines. **a** Proliferation of PDS models treated with palbociclib 1 µM, palbociclib STC or 0.01% DMS0 (vehicle). **b** Proliferation of PDR models treated with palbociclib 1 µM or 0.01% DMS0 (vehicle). Growth rate was assessed at day 6 by methylene blue assay. Growth fold changes were computed as ratio, dividing growth rate of treated cells by their controls treated with drug vehicle. Mean value of growth fold change of three independent experiments ±standard error of the mean (SEM) was plotted (*P-values < 0.05, two–way ANOVA with Sidak’s multiple comparisons test). **c** β-galactosidase activity of PDS and PDR models treated with palbociclib 1 µM or 0.01% DMS0 (vehicle). Positive and negative cells were counted in six randomly selected 20X fields and the percentage of positive cells was calculated. Mean percentage value of β-galactosidase positive cells of three independent experiments ±SEM was plotted (*P-values < 0.05, by Student’s *t* test)

Proliferation of all PDS cells was significantly inhibited by palbociclib (*P*-value < 0.05), while PDR derivatives did not show significant differences in cell growth when treated with palbociclib 1 µM compared to vehicle-treated cells (Fig. 1a,b). Additionally, PDR cells showed IC50 values 6–30 times higher than their PDS counterparts (Supplementary Table s1). CDK4/6 inhibitors are cytostatic drugs, which induce a senescence-like phenotype, typically characterized by high β-galactosidase expression and a flattened cellular shape.^11^ With the exception of the MDA MB 361 cell line, our PDR models showed significantly (*P*-value < 0.05) lower proportions of β-galactosidase positive cells during palbociclib treatment compared to their PDS counterparts, demonstrating the ability to revert the palbociclib-induced senescence-like phenotype (Fig. 1c and Supplementary Fig. s1).

### Gene expression (GE) analysis of PDS and PDR cells reveals that modulation of E2F signaling occurs during palbociclib treatment and at the time of acquired resistance in HR+ BC cell lines

We analyzed GE data derived from PDS cells treated with drug vehicle (control, PDS) or with palbociclib starting treatment concentration (STC) for 3 days (PDS treated) and from PDR cells continuously receiving palbociclib 1 µM.

**Fig. 2:**
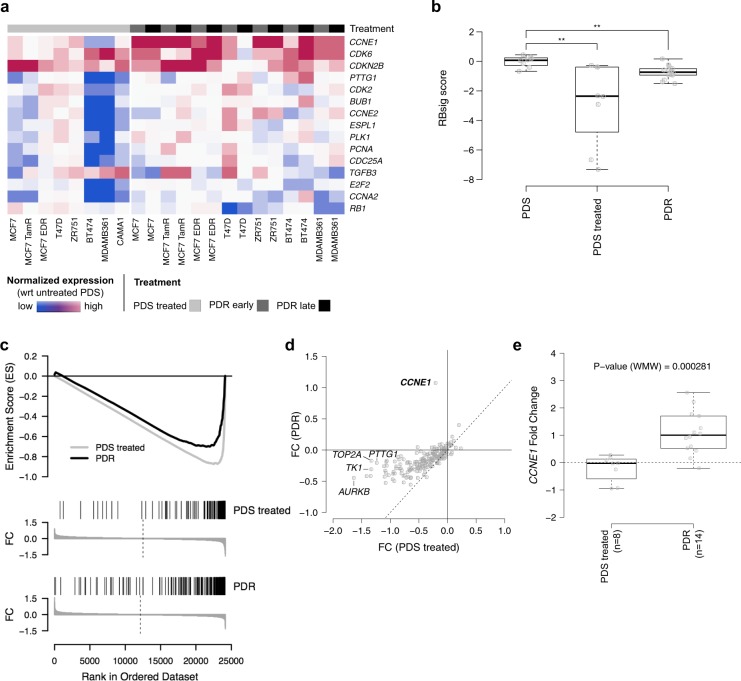
GE analysis of PDS and PDR cells. **a** Heatmap showing the top 15 most variable cell cycle pathway genes (rows) between PDR and PDS treated cells (columns). Values are the log2 of Fold Change (FC) of normalized expression in each cell lines with respect to the corresponding PDS. The different cell models are reported in the annotation track (PDS treated, PDR early, PDR late). **b** Box plot showing the distribution of RBsig score across the different cell models. **c** Combined Gene Set Enrichment Analysis (GSEA) plot of PDS treated (gray lines) and PDR (black lines) using averaged FC as ranking parameters and E2F targets as gene set. For both PDS treated and PDR, FDR ~ 0. **d** Averaged FC in PDR (y-axis) versus averaged FC in PDS treated for E2F target genes. The top five genes having the greatest absolute difference in FC between PDR and PDS treated models are shown. **e** Box plot showing the distribution of FC of *CCNE1* expression in PDS treated and PDR models. **P-value < 0.01, ***P-value < 0.001; by Wilcoxon test

Hierarchical clustering analysis of GE profiles did not reveal common GE programs associated with palbociclib resistance; conversely, cells tended to segregate based exclusively on their cell type (Supplementary Fig s2), indicating that common characteristics at the time of resistance were not able to overcome cell-type specific features. To reduce cell-type specific signals, we performed an intra-cell type normalization of GE and calculated differential expression between PDR and PDS treated cells. The list of the top 100 differentially expressed genes is provided in Supplementary Table s2. None of the differentially expressed genes were significantly over or underexpressed when adjusted for multiple testing (Supplementary Table s2), probably due to the high heterogeneity observed among different cell lines. However, GE analysis of the cell cycle pathway revealed that *CCNE1* and *RB1* were consistently deregulated in all PDR models versus PDS treated cell lines (Fig. 2a). This prompted further analyses of the Rb/E2F pathway during palbociclib treatment and at the time of resistance in our models.

We first analyzed a previously developed GE signature of Retinoblastoma loss-of-function, the RBsig,^12^ in PDS, PDS treated and PDR models. Overall, RBsig levels were significantly reduced by palbociclib treatment in PDS treated cells (Fig. 2b). Such modulation differed depending on the cell type, with the MDA MB 361 and BT474 HER2+ cell lines showing the highest levels of downregulation (Supplementary Fig. s3). In PDR models, RBsig levels tended to return to basal levels, but remained significantly inhibited compared to PDS untreated cells (Fig. 2b).

To further investigate the role of Rb/E2F pathway during palbociclib treatment and at the time of resistance, we performed GSEA on the normalized GE levels using the “HALLMARK_E2F_TARGETS” gene set, comprising 200 genes, of which 186 were present in our expression data. Consistently with RBsig analysis, genes down-regulated during treatment with palbociclib were significantly enriched for E2F targets; (FDR~0, Enrichment Score, ES = −0.88); similarly, genes down-regulated at the time of resistance were also significantly enriched in E2F targets indicating that the pathway remains inhibited by palbociclib at the time of resistance (FDR~0, ES = −0.71) (Fig. 2c). Interestingly, only *CCNE1* showed a significant increase of expression at the time of resistance (*P*-value < 0.0005) (Fig. 2d,e), while the majority of the E2F target genes tended to be down-regulated both during treatment and at the time of resistance (Fig. 2d).

Overall these data suggest that palbociclib effectively inhibits cell growth of HR+ BC cells through inhibition of the Rb/E2F pathway. However, at the time of resistance, signaling is only partially restored, with increased *CCNE1* expression being a common event.

### Analysis of cell cycle-related proteins in PDS and PDR cells confirms that alterations of cyclin E1 and Rb expression are common events at the time of palbociclib acquired resistance

To validate the role of cell cycle-related proteins during palbociclib treatment and at the time of resistance, we performed western blot (WB) analysis for selected markers in PDS cell lines treated with palbociclib for 3 and 6 days at the STCs, and in PDR models continuously receiving palbociclib 1 μM.

**Fig. 3:**
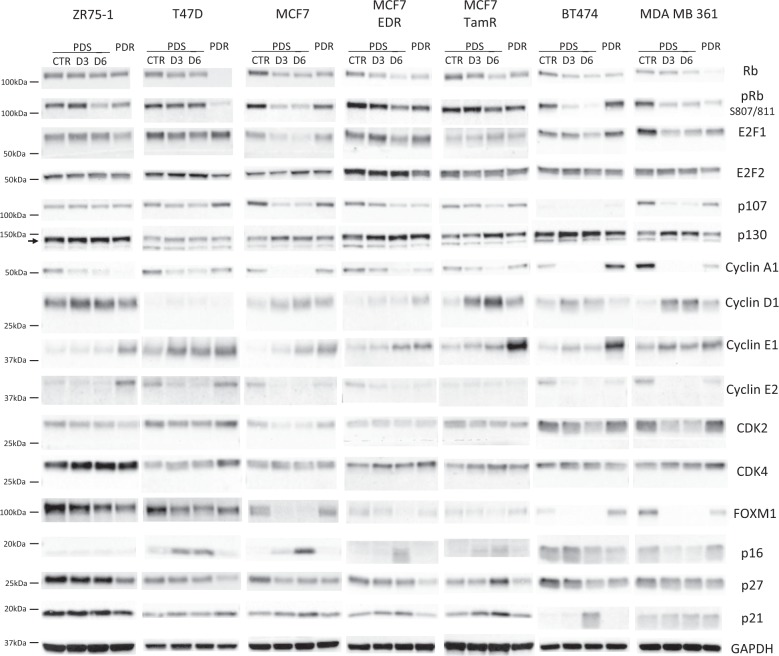
Analysis of cell cycle-related proteins in PDS and PDR cells. Western blot analysis of cell lysates from PDS models treated with vehicle (CTR) or palbociclib at the STCs for 3 and 6 days (D3, D6) and from their PDR counterparts receiving palbociclib 1 μM. Samples were blotted with the indicated antibodies. GAPDH bands are representative of one blot for each cell line. Blots from a given cell line (PDS and PDR) derive from the same experiment and were processed in parallel. Uncropped images of the cyclin E1 and Rb blots are supplied as Supplementary Figure s7

Overall, different cell cycle-related proteins showed a similar modulation in all models during palbociclib treatment, although extent of modulations differed among cell lines. As expected, we observed a consistent reduction of Rb and its phosphorylated Ser 807/811 form after 3–6 days of treatment (Fig. 3). Similarly, E2F1 was generally down-regulated during palbociclib treatment together with the Rb related protein p107, cyclin A1 and cyclin E2 (Fig. 3). Additionally, levels of the transcription factor FOXM1, a potential target of CDK4/6 signaling,^13,14^ were consistently downregulated by palbociclib. Conversely, cyclin D1 and cyclin E1 levels tended to increase during treatment. All other markers were modulated heterogeneously, or were not modulated by the treatment (Fig. 3).

When PDR cells were compared to their PDS counterparts, in accordance with the GE data, we found significant heterogeneity in the regulation of the majority of cell cycle-related proteins among models. Very few modulations were consistent in all cell lines. Compared to untreated PDS cells, Rb was downregulated in all PDR models, with T47D showing virtually complete loss of expression (Fig. 3). Similarly, phosphorylation of Rb was reduced in PDR compared to untreated PDS cells (Fig. 3). Additionally, the cell cycle inhibitor p27 showed reduced expression in PDR compared to PDS cells lines (Fig. 3), whereas cyclin E1 was consistently upregulated in all models (Fig. 3), confirming the GE observation. All other cell cycle-related proteins were differently regulated in each PDR model or not modulated (Fig. 3). Quantification of the bands for Rb and cyclin E1 is provided as supplementary table s3.

### Genomic analysis of *CCNE1* and *RB1*

**Fig. 4:**
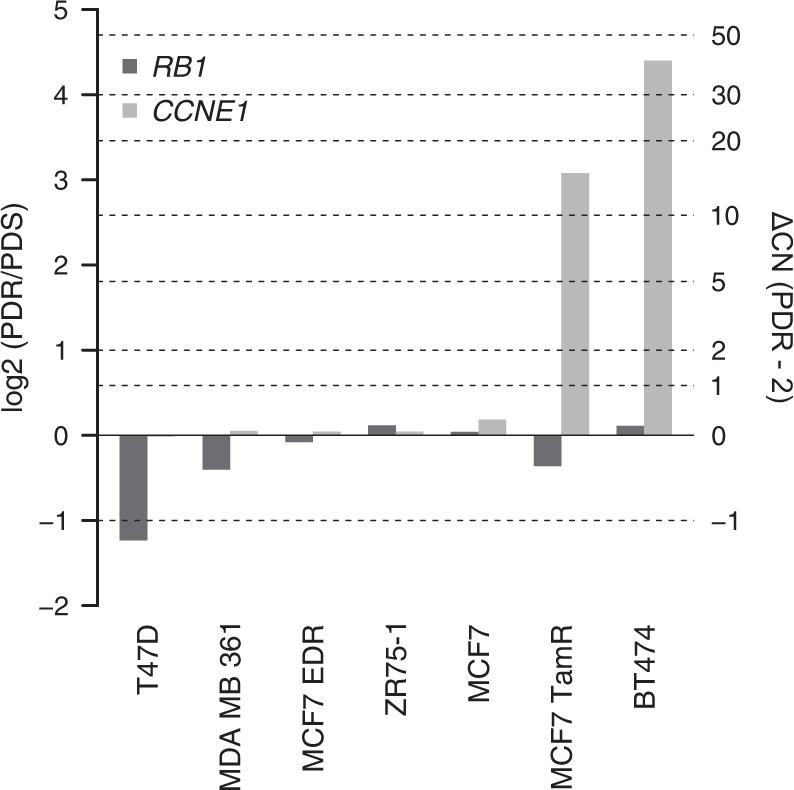
Genomic analysis of *CCNE1* and *RB1*. Bar plot showing the log2 of the ratio of copy number estimates for *RB1* (dark gray) and *CCNE1* (gray) in PDR to untreated PDS models. Right vertical axis reports the estimated copy number difference between PDR and untreated PDS models, assuming 2 copies for PDS cells

A potential mechanism for cyclin E1 and Rb deregulation could be represented by genomic alterations encompassing the corresponding genes. We therefore analyzed the copy number status of *CCNE1* and *RB1*, normalized to the reference gene *EIF2C1*, in all PDR and PDS models. BT474 and MCF7 TamR exhibited an increased copy number status of *CCNE1*, while T47D, MDA MB 361 and MCF7 TamR showed reduced copy number of *RB1* at the time of resistance (Fig. 4). Indeed, the log2 of the ratios of *CCNE1/EIF2C1* between the BT474 and TamR PDR cells and PDS counterparts were 4.4 and 3.08, respectively. Interestingly, these data suggest that >30 and >10 *CCNE1* DNA copies are acquired at resistance in BT474 and TamR PDR cells. Conversely, the log2 of the ratios of *RB1*/ *EIF2C1* in T47D, MDA MB 361, and MCF7 TamR PDR cells were −1.24, −0.40, and −0.36, respectively, indicating a genomic loss of *RB1* in these cell lines.

### *CCNE1* and *CCNE1*/*RB1* ratio as markers of de novo resistance to palbociclib

**Fig. 5:**
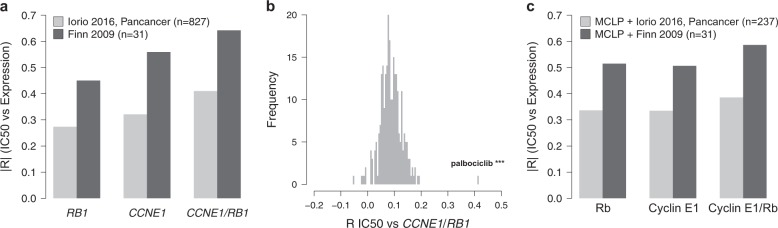
*CCNE1* and *CCNE1*/*RB1* ratio as markers of de novo resistance to palbociclib. **a** Bar plot of the absolute value of Pearson’s correlation coefficient of IC50 values versus expression of *RB1*, *CCNE1*, and *CCNE1*/*RB1* by considering both IC50 values and expression data for 827 cell lines of different tumors from Iorio et al., 2016^20^ (light gray); IC50 values and expression data for 31 BC cell lines from Finn et al., 2009^19^ and Iorio et al., 2016^20^, respectively (dark gray). **b** Histogram of the absolute value of Pearson’s correlation coefficient of *CCNE1*/*RB1* versus IC50 values for 265 compounds tested in the cancer cell line dataset of Iorio et al.^20^ Palbociclib is the compound showing the most significant association with *CCNE1*/*RB1*. ***P < 0.001; by correlation test. **c** Bar plot of the absolute value of Pearson’s correlation coefficient of IC50 values versus expression of Rb, Cyclin E1, and Cyclin E1/Rb by considering IC50 values and protein expression data for 237 cell lines of different tumors from Iorio et al.,^20^ and MCLP^21^, respectively (light gray); IC50 values and expression data for 31 BC cell lines from Finn et al.^19^, and MCLP^21^, respectively (dark gray)

Our results demonstrated that cyclin E1 overexpression and Rb downregulation are common findings at the time of acquired resistance to palbociclib. The role of Rb in determining de novo resistance to CDK4/6 inhibitors has already been implicated in BC.^15–19^ Here we aimed to investigate if *CCNE1* and the combined analysis of *CCNE1* and *RB1* as *CCNE1*/*RB1* ratio could also represent a marker of de novo resistance to palbociclib. We therefore correlated *CCNE1* and *RB1* expression levels and *CCNE1*/*RB1* ratio with palbociclib IC50 in a large dataset of cancer cell lines.^20^ In cell lines derived from different cancer types (pan-cancer dataset), these markers significantly correlated with higher IC50 values for palbociclib (Fig. 5a light gray bars, minimum |R| >0.27, maximum FDR = 5*10^−14^, correlation test) and among all drugs tested in the dataset^20^ palbociclib showed the strongest association with *CCNE1*/*RB1* ratio (Fig. 5b, *R* = 0.41, FDR <10^−30^, correlation test). In addition, analyzing GE data from BC cell lines of the Iorio et al. dataset,^20^ but taking into account the IC50 values for palbociclib obtained from a previous BC-focused study,^19^ we were able to demonstrate that *CCNE1, RB1*, and *CCNE1* /*RB1* ratio correlated with higher IC50 also in BC cell lines (Fig. 5a dark gray bars, minimum |R| >0.45, maximum FDR = 0.01, correlation test). Of note, both in the pan-cancer and in the BC datasets, *CCNE1*/*RB1* performed better than *CCNE1* or *RB1* alone in predicting palbociclib de novo resistance (Fig. 5a).

The correlations between protein levels of Rb1, cyclin E1, cyclin E1/Rb ratio, and palbociclib IC50 were also tested in a large dataset of cancer cell lines analyzed by reverse-phase protein arrays.^21^ Rb, cyclin E1, and the cyclin E1/Rb ratio correlated with palbociclib IC50, with the cyclin E1/Rb ratio performing better than cyclin E1 and Rb alone in predicting palbociclib resistance, both in the pan-cancer and the BC datasets (Fig. 5c).

### *CCNE1*/*RB1* ratio is a poor prognostic factor and a marker of palbociclib resistance in estrogen receptor-positive (ER+) BC

**Fig. 6:**
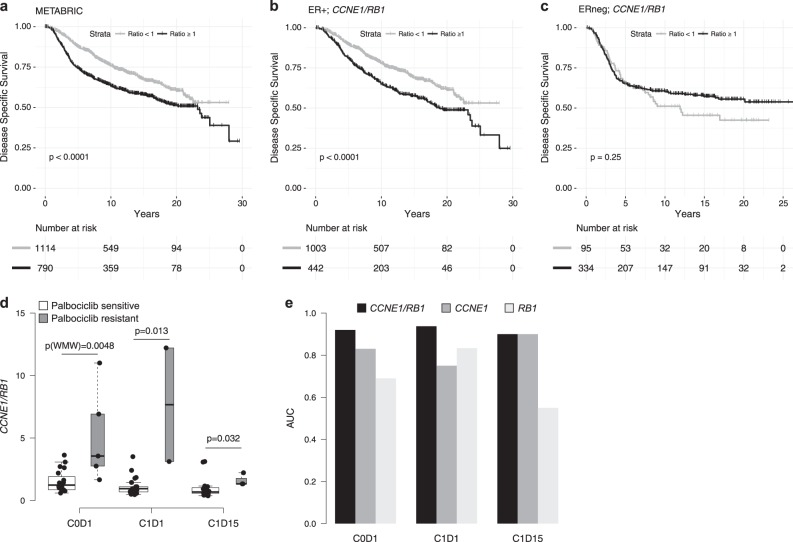
*CCNE1*/*RB1* ratio as a poor prognostic factor and a marker of palbociclib resistance. **a** Kaplan–Meyer plot of METABRIC dataset^22^ (*n* = 1904). **b** Kaplan–Meyer plot of METABRIC ER+ samples (*n* = 1445). **c** Kaplan–Meyer plot of METABRIC ERneg samples (*n* = 429). Samples are divided into two groups according to the ratio of *CCNE1* to *RB1* expression. Cut-off is set to 1. *P*-values are calculated with log-rank test. **d** Box plot of the distributions of the ratio between gene expression level of *CCNE1* to *RB1* expression (*CCNE1/RB1*) in the NeoPalAna dataset^23^ for Palbociclib-sensitive (white) and resistant (gray) patients at the different time points. *P*-values are calculated by Wilcoxon–Mann–Whitney (WMW) test. **e** Bar plot showing the *CCNE1/RB1* (dark gray), *CCNE1* (gray), and *RB1* (light gray) AUC values for discriminating between palbociclib-sensitive and resistant patients at the different time points

The prognostic role of the *CCNE1*/*RB1* ratio in ER+ BC has previously never been investigated. As such, we analyzed survival of patients included in the METABRIC dataset^22^ according to the *CCNE1*/*RB1* ratio. In the overall population, a significantly poorer overall survival (OS) was found in patients with a higher *CCNE1/RB1* ratio (*P*-value < 0.0001, Fig. 6a). In order to exclude the potential confounder that the observed poor survival could be related to the association of the *CCNE1*/*RB1* ratio with molecular subtypes (Supplementary Fig. s4), survival of the ER+ and ER negative (ERneg) subpopulations was analyzed separately. Interestingly, in the ER+ population the prognostic role of the *CCNE1*/*RB1* ratio was maintained, with patients with high ratio (≥1) showing a poorer survival compared to those with a ratio <1 (*P*-value <0.0001, HR = 0.63, Fig. 6b). Conversely, in the ERneg population we observed no effect of the *CCNE1*/*RB1* ratio on survival (*P*-value = 0.25, HR = 0.82, Fig. 6c). Results were similar when the median value of *CCNE1*/*RB1* ratio in the ER+ population was used as cut-off (Supplementary Fig. s5a, b).

Further validation was sought through analysis of the publicly available NeoPalAna dataset. In the NeoPalAna trial, patients with stage II/III ER+/HER2neg primary BC received neoadjuvant palbociclib and anastrozole.^23^ Serial biopsies performed at baseline (C0D1), before starting palbociclib (C1D1) and after 2 weeks of palbociclib (C1D15), and surgical samples were analyzed for Ki67, GE, and mutation profiles.^23^ We found that *CCNE1/RB1* was significantly higher in patients resistant to palbociclib at C0D1, C1D1, C1D15 (p(WMW) = 0.0048; *p* = 0.013; *p* = 0.032, respectively, Fig. 6d). Also, *CCNE1/RB1* performed better than *CCNE1* and *RB1* alone in discriminating palbociclib-resistant versus sensitive patients at C0D1 (AUC(*CCNE1/RB1*) = 0.92; AUC(*CCNE1*) = 0.83; AUC(*RB1*) = 0.69), and at C1D1 (AUC(*CCNE1/RB1*) = 0.92; AUC(*CCNE1*) = 0.75; AUC(*RB1*) = 0.83), while showing equivalent performance compared to *CCNE1* at C1D15 (AUC(*CCNE1/RB1*) = 0.9; AUC(*CCNE1*) = 0.9; AUC(*RB1*) = 0.55) (Fig. 6e).

## Discussion

In this study, we developed a panel of HR+ BC cell lines with acquired resistance to palbociclib by chronically exposing cells to increasing doses of the drug. To account for the heterogeneity of luminal BC, we selected eight BC cell lines with different initial degrees of sensitivity to palbociclib. We deliberately challenged cell lines to palbociclib alone, rather than combined long-term estrogen deprivation (LTED) and palbociclib in order to identify markers specifically related to palbociclib resistance, thus avoiding the confounding effect of endocrine treatment. We acknowledge that the resultant models might not fully recapitulate the clinical scenario wherein CDK4/6 inhibitor resistance occurs in the setting of dual hormonal therapy and palbociclib. However, we did include two models previously resistant to hormonal therapy in order to account for the effect of combined palbociclib and endocrine resistance. Two HR+/HER2+ cell lines were also included to search for strong common mechanisms of resistance that might occur in HR+ BC despite HER2 status, given that palbociclib has also shown efficacy in HER2+ BC models.^19,24–26^ We obtained reliable models of acquired resistance to palbociclib, as revealed by functional analyses investigating proliferation, IC50 and β-galactosidase expression, for all cell lines except CAMA1. Yang et al. succeeded in the development of models of CAMA1 resistant to abemaciclib,^10^ probably due to the different approach used^10^ and the different inhibitory profile of abemaciclib.^27^

As a first step, we explored the effects of palbociclib on the Rb/E2F pathway, which is the target of CDK4/6 inhibitors.^28,29^ As expected, and in line with previous reports,^9,30,31^ we observed an inhibition of this pathway in response to palbociclib. We observed a marked reduction of the RBsig, a gene-signature of loss of *RB1* function^12^ and found an enrichment in E2F targets within genes downregulated by palbociclib, consistent with previous observations.^30^ The activity of palbociclib on the Rb/E2F pathway was confirmed by WB, as revealed by decreased levels of total Rb coupled with decreased phosphorylation of Rb on S807/811 after 3 and 6 days of treatment. Together with Rb, we also observed a general decrease in p107 levels in all cell lines. As already reported by previous studies with cell lines sensitive to CDK4/6 inhibitors,^9,31^ our PDS models displayed cyclin D1 accumulation, reduction of E2F-regulated proteins (such as cyclins A1 and E2 and CDK2), and p130 stabilization when receiving palbociclib.

It has been demonstrated that BC cells show adaptation following CDK4/6 inhibition as early as 72 h. This has been shown to be mediated by non-canonical activation of cyclin D1/CDK2 complexes, which in turn induce a recovery of Rb phosphorylation and S-phase entry despite CDK4/6 inhibition.^9^ Our PDS models showed a persistent inhibition of the Rb/E2F pathway after 3 and 6 days of treatment. However, we did not analyze earlier time points, and therefore cannot exclude a partial recovery of the pathway at 72 h as suggested by Herrera-Abreu et al.^9^

It has already been shown that alterations in cell cycle genes and proteins are associated with acquired resistance to palbociclib.^8–10,32,33^ In particular, cyclin E overexpression and Rb loss have been previously indicated as possible mechanisms of acquired resistance to CDK4/6 inhibitors in some BC cell lines.^8–10^ Our GE and WB data showed that both cyclin E1 overexpression and Rb underexpression occur across different and heterogeneous BC cellular models, including HER2+ cells, which has not been shown previously. We also demonstrated that *CCNE1* amplification and *RB1* deletion might explain only partially these findings, suggesting that other mechanisms could be implicated. Intriguingly, these genomic alterations occurred in both HER2+ and HER2neg cell lines, again supporting the hypothesis that strong mechanisms of acquired resistance to palbociclib might occur in HR+ BC independent of HER2 status. This is of potential clinical relevance as CDK4/6 inhibitors are currently under investigation for patients with HR+ /HER2+ BC.^34^

At the time of resistance, we observed a slight reactivation of the Rb/E2F pathway as demonstrated by the increased RBsig expression in PDR cells, and by the modulation of some of the E2F targets analyzed. However, the pathway remained significantly inhibited compared to the PDS untreated cells, indicating that palbociclib treatment may still be inhibiting the pathway. This is consistent with previously published data in ovarian cancer, wherein cells resistant to palbociclib did not fully restore E2F target gene expression.^32^

Another common marker that we found at the time of acquired resistance is the reduced expression of p27. In a previous study, Dean et al. similarly suggested that loss of CDK inhibitors p21 and p27 may foster the ability of cells to escape from palbociclib-induced cell cycle arrest.^31^ Intriguingly, phosphorylation status of p27 has also been suggested as a mechanism of resistance to CDK4/6 inhibitors.^35^

Clinical trials with palbociclib clearly show that some patients do not benefit from CDK4/6 inhibitors.^2–4,36^ Loss of *RB1* has been already suggested as a mechanism of de novo resistance to these drugs.^15–19^ Here, we identified *CCNE1* as an additional potential marker of de novo resistance in a large panel of pan-cancer and BC cell lines. To date, the role of cyclin E1 as a marker of de novo resistance has been demonstrated in other tumor types such as ovarian and gastric cancer^37,38^ but only suggested in BC.^33,39^ Our data suggest that both *CCNE1* and the *CCNE1/RB1* ratio, which evaluates both *CCNE1* and *RB1*, might serve as “pan-cancer” markers of de novo resistance to palbociclib and that the *CCNE1*/*RB1* ratio might serve as better marker compared to the single biomarkers *CCNE1* and *RB1* used separately, both in BC and in other cancer types. In BC, this is evident for palbociclib IC50 values from Finn et al,^19^ but not when values from Iorio et al.^20^ were considered (Fig. 5a, c and Supplementary Fig. s6a, b). This discrepancy may be attributed to the different methodologies and drug concentrations used in the two studies,^19,20^ and might impact to a lesser extent when a larger number of heterogeneous pan-cancer cell lines are used, explaining the ability to reveal the correlations between *CCNE1*, *CCNE1*/*RB1* ratio, and palbociclib IC50 when analyzing the pan-cancer dataset.

One limitation of our study is the lack of clinical validation of our findings on the role of *CCNE1* and *RB1* as markers of acquired resistance to palbociclib. Ideally, this should be evaluated prospectively in tissue biopsies obtained at the time of disease progression on palbociclib treatment, which was not performed as a part of this study. In lieu, we sought validation of our preclinical findings regarding *CCNE1*/*RB1* ratio as a marker of de novo resistance to palbociclib in the NeoPalAna trial.^23^ Results from the NeoPalAna trial already showed that *CCNE1* expression was significantly elevated in tumors resistant to the combination of palbociclib and anastozole at C1D15.^23^ We demonstrated that the *CCNE1*/*RB1* ratio was significantly higher in palbociclib-resistant tumors at all time points analyzed. Of note, *CCNE1*/*RB1* performed better than *CCNE1* and *RB1* alone in predicting de novo resistance to palbociclib at C0D1 and C1D1. These findings hold additional interest in light of the recently published TREnd trial,^40^ a phase 2 clinical study which assessed the activity of palbociclib as monotherapy, as well as in combination with the endocrine therapy (aromatase inhibitor or fulvestrant) upon which the patient had experienced previous progression as a prior line of therapy for ER+/HER2neg metastatic BC. This might be a potential additional setting to validate the role of the *CCNE1/RB1* as a marker of de novo resistance to palbociclib.

Recent data have shown that expression of the cytoplasmic low-molecular-weight isoforms (LMW) of cyclin E is associated with resistance to aromatase inhibitors^41^ and to palbociclib in vitro.^42^ Similarly, overexpression of LMW cyclin E detected by immunohistochemistry has been associated with a lesser benefit from treatment with palbociclib and endocrine therapy in patients with metastatic BC.^42^ In our WB studies of the PDR models, LMW isoforms of cyclin E1 were not detected, suggesting that the full length is predominantly overexpressed in our models of acquired resistance. However, our in silico analyses could not exclude the possibility that LMW cyclin E may contribute to the effect on de novo resistance we described for cyclin E1, as both RNA and RPPA data cannot distinguish between the full length and the LMW isoforms.

We demonstrated that patients with early ER+ BC with a high *CCNE1*/*RB1* ratio have poorer OS compared to patients with a ratio <1, suggesting that this may represent a population of patients with an adverse outcome, which might mandate tailored therapeutic strategies. However, more detailed studies are necessary to dissect the prognostic versus predictive value of this biomarker in early BC, which would be of particular interest since CDK4/6 inhibitors are currently being tested in the adjuvant setting.^43^

The strengths of our study include the size of the employed cell lines panel: to the best of our knowledge, this is the largest panel of HR+ BC cell lines ever developed to investigate acquired resistance to palbociclib. In turn, we have expanded previous knowledge on acquired resistance to palbociclib by confirming *CCNE1* overexpression/amplification and reduced expression/loss of *RB1* as common events that occur at the time of acquired resistance in luminal BC cell lines, independent of HER2 status. Furthermore, we have identified the *CCNE1*/*RB1* ratio as a potential marker of de novo resistance to palbociclib both in vitro and in clinical samples.

Results of our study, if further confirmed and tested in clinical trials, might lead to the identification of new biomarkers of de novo and acquired resistance to palbociclib, which would assist to better personalize treatment of patients with HR+ BC.

## Methods

### Cell lines, cell culture, and reagents

T47D and ZR75-1 cell lines were obtained from Dr Livia Malorni, CNR Avellino, Italy; CAMA1 were obtained by American Type Culture Collection; MCF7 parental cells, MCF7 EDR and MCF7 TamR were previously described.^44^ MDA MB 361 were purchased from Sigma-Aldrich and BT474 from Interlab Cell Line Collection, Genova, Italy.

Cells were cultured at 37 °C and 5% CO2. T47D, ZR75-1, MCF7, CAMA1, MDA MB 361, and BT474 cells were grown in Dulbecco’s modified Eagle’s medium (DMEM) with 4.5 g/glucose and L-glutamine (Lonza) supplemented with 10% heat-inactivated fetal bovine serum (FBS) (Hyclone) and, 10,000 U penicillin and 10 mg steptomycin/mL solution (P/S) (Sigma-Aldrich). MCF7 EDR and MCF7 TamR were grown in DMEM with 4,5 g/glucose and without L-glutamine and phenol red (Lonza) supplemented with 10% charcoal-stripped FBS (CS-FBS) (GIBCO), and P/S. MCF7 TamR cell medium was supplemented with 100 nM final concentration of (Z)−4-Hydroxytamoxifen (Sigma-Aldrich) dissolved in 100% ethanol.

PDR were generated by long term culturing of cells with increasing concentrations of palbociclib from the STC up to 1 μM. STC was preliminary assessed on each model and defined as the concentration that, after 9 days of treatment, inhibited cells growth rate between 40 and 60%. Cells were considered resistant to palbociclib when they restored the ability to regularly grow in the presence of palbociclib 1 μM.

Palbociclib STC was 50 nM for T47D, ZR75-1, MCF7 EDR, and MCF7 TamR cell lines, 100 nM for CAMA1 and 350 nM for MCF7, MDA MB 361 and BT474 cell lines. PDR cells were then continuously cultured in their original media with the addition of palbociclib 1 μM. Palbociclib was provided by Pfizer and dissolved in dimethyl sulfoxide (DMSO) (Sigma-Aldrich).

In January 2016, all cell lines and their PDR derivatives have been authenticated by short tandem repeat DNA profiling analysis. This analysis was performed in service by BMR Genomics, Padova, Italy.

### Proliferation and IC50 assays

For proliferation and IC50 assays a total of 3000 cells/well of PDS or PDR cell lines, cultured in their individual media, were plated in 96-well plates in triplicate, 24 h before beginning treatments. These consisted of palbociclib 1 µM, palbociclib STC or 0.01% DMS0 (vehicle) for 6 days for proliferation assays or doubling dilutions of palbociclib, from 64 µM to 0.97 nM for 9 days for the IC50 assays. Media were replaced every 72 h. Cells were fixed with 4% glutaraldehyde grade II (Sigma-Aldrich) and stained with 0.05% methylene blue (Sigma-Aldrich). The dye was subsequently extracted with 3% HCl (Carlo Erba) and absorbance measured at 655 nm. Three biological replicates for each PDS or PDR cell line were performed.

### β-galactosidase assay

PDS and PDR cell lines were plated in 12-well plates in duplicate, in a range of 10,000–20,000 cells/well. Cells were plated in their individual medium which was replaced after 24 h with medium containing palbociclib 1 µM or 0.01% DMSO as a control. Beta-galactosidase activity was assessed at day 6 by Senescence detection kit (BioVision) according to the manufacturer’s instructions. Positive and negative cells were counted in six randomly selected 20X fields and the percentage of positive cells was calculated. Three biological replicates for each PDS or PDR line were performed.

### Nucleic acids isolation and gene expression profiles

DNA was isolated from PDS and PDR cells grown in their individual media supplemented with 0.01% DMSO or palbociclib 1 µM, respectively. DNA extraction was performed with the Quick-gDNA^TM^ MiniPrep kit (Zymo Research), according to the manufacturer’s instructions. DNA concentrations and quality were checked by spectrophotometer.

RNA was isolated from PDS cells treated with 0.01% DMSO, as a control, or with palbociclib at the STC for 3 days, and from PDR cells at early (before or at passage 15) or late (after passage 15) stage of resistance, grown in their individual media supplemented with palbociclib 1 µM. The number of passages of PDR cells was calculated starting from the moment they restored the ability to regularly proliferate in the presence of palbociclib 1 µM. RNA was extracted from cells with the Direct-zol^TM^ RNA MiniPrep kit (Zymo Research) according to the manufacturer’s instructions. RNA quantification and quality analysis were performed on a Bioanalyzer 2100 System (Agilent), using the RNA 6000 nano Kit (Agilent). cDNA synthesis and biotin-labeled cRNA generation were performed using the Illumina TotalPrep RNA Amplification Kit (Ambion), according to the manufacturer’s protocol using 500 ng of total RNA. Quality assessment and quantification of cRNAs were performed with Agilent RNA kits on the Bioanalyzer 2100 System. Hybridization of cRNAs (750 ng) was carried out using Illumina Human 48k gene chips (Human HT-12 v4 BeadChip). Array washing was performed using Illumina High Temp Wash Buffer for 10′ at 55 °C, followed by staining using streptavidin-Cy3 dyes (Amersham Biosciences). Probe intensity data were obtained using the Illumina GenomeStudio software.

### GE data analysis

Normalization of probe intensity data (normalized expression) was performed by cubic spline algorithm implemented in the Illumina GenomeStudio software version 1.9.0. Only probes showing a detection *P*-value < 0.05 in at least one sample were considered for downstream analysis (*n* = 31016). In case of multiple probes matching to a single gene, the probe with the highest signal-to-noise ratio was taken into consideration. To mitigate the effect of cell line specific expression profiles, the log2-ratio values of normalized expression of treated PDS and PDR to the corresponding untreated PDS GE profiles were computed. (Fold Change, FC). The list KEGG_CELL_CYCLE (http://software.broadinstitute.org/gsea/msigdb/cards/KEGG_CELL_CYCLE) was used to identify cell cycle pathway genes. Top variable cell cycle genes (Fig. 2a) were defined as 25% most variable cell cycle genes across treated PDS and PDR (i.e., by standard deviation of FC) having an absolute value of average FC in PDR greater than 0.2, resulting in 15/127 genes.

E2F gene set enrichment was computed with Gene Set Enrichment Analysis (GSEA) version 2-2.2.3 by using log2-ratio values of averaged (mean) FC across PDS treated and PDR cell line models as ranking parameters and HALLMARK_E2F_TARGETS (http://software.broadinstitute.org/gsea/msigdb/cards/HALLMARK_E2F_TARGETS) as gene set. Differential expression between log2-ratio expression values was tested by Wilcoxon–Mann–Whitney (WMW) test.

### Evaluation of RBsig score

For each RBsig^12^ gene *i* = *1,.,N*, the median $\left( {\overline {g_{i,{\mathrm{PDS}}}} } \right)$ and median absolute deviation ${\mathrm{mad}}(g_{i,{\mathrm{PDS}}})$ of normalized expression in untreated PDS cell lines were estimated. RBsig score of each cell line *j* was then calculated by the following formula:

$${\mathrm{RBsig}}_j = \frac{{\mathop {\sum}\limits_i {\frac{{g_{ij} - \overline {g_{i,{\mathrm{PDS}}}} }}{{{\mathrm{mad}}(g_{i,{\mathrm{PDS}}})}}} }}{N},$$

where *g*_*ij*_ is the normalized expression of RBsig gene *i* in the *j*-th cell line.

### Western blot

PDS cells treated with 0.01% DMSO, as a control, or with the STC of palbociclib for 3 or 6 days and PDR cells grown in their individual media supplemented with palbociclib 1 µM were lysed with RIPA buffer (Cell Signaling Technology), supplemented with 1% Protease Inhibitor Cocktail (Sigma-Aldrich) and 1% Halt^TM^ Phosphatase Inhibitor Cocktail (Thermo Scientific). A volume of 15 μg of proteins were separated under denaturing conditions by electrophoresis on 10% or 7.5% polyacrylamide gels (TGX Stain-Free^TM^ Gels, Bio-Rad) and transferred to nitrocellulose membranes (Trans-Blot Turbo® Transfer Pack^TM^, 0,2 µm nitrocellulose, Bio-Rad) by electroblotting (Trans-Blot® Turbo^TM^ Transfer System, Bio-Rad). The blots were checked using the ChemiDoc MP Imaging System and the Image Lab software (Bio-Rad) to confirm uniform transfer of all samples. Blots were then blocked with Tris-buffered saline (Sigma-Aldrich) containing 0.1% Tween 20 solution (Sigma-Aldrich) and 5% non-fat dry milk (Cell Signaling Technology) or 5% BSA (Santa Cruz Biotechnology) for 1 h at room temperature (RT) and then incubated with primary and secondary antibodies.

Analysis of WB bands and normalization to total proteins were performed using the Image Lab^TM^ software v 5.2.1 (Bio-rad). WB were repeated in two biological replicates. A list of primary and secondary antibodies with their working conditions is provided in Supplementary Table s4.

### *RB1* and *CCNE1* CNV analysis by ddPCR

Copy number alterations of *RB1* and *CCNE1* between PDR and PDS cells were assessed with *RB1*, *CCNE1* and the reference gene *EIF2C1* CNV Assays on a QX200^TM^ droplet digital PCR (ddPCR) system (Bio-Rad). PCR reactions were prepared with approximately 10 ng of DNA and ddPCR supermix for probes (Bio-Rad). *RB1* and *CCNE1* probes (Bio-Rad) were stained in FAM while *EIF2C1* probe (Bio-Rad) was stained in HEX. The ratios *CCNE1*/*EIF2C1*and *RB1*/*EIF2C1* were calculated using Quantasoft software (Bio-Rad). At least one negative control with no DNA was included in each run. Experiments were performed three times in duplicate.

### Statistical analysis

For proliferation assays, growth fold changes were computed as ratio, dividing growth rate of treated cells by their controls treated with DMSO. Mean value of growth fold change of all experiments ±standard error of the mean (SEM) was plotted. Two–way ANOVA with Sidak’s multiple comparisons test was performed with GraphPad Prism version 7.03 and *P-*values < 0.05 were considered significant.

For IC50, the signals were converted to proportions adjusting on time zero (T0) and on untreated DMEM control values and fitted using the 4-parameter logistic regression (R package nplr version 0.1–4). The proportion values, *yp*, were computed as: *yp* = (*y* − T0)/(DMSO − T0), where *y* are the observed values. The model performance was estimated by a weighted standard error, and a weighted goodness-of-fit. Once the model was fitted, the drug 50% inhibitory concentration (IC50) could be estimated from inhibition rates by inverting the function (given a *y* value = 0.5 survival rate). Mean value of IC50 of all experiments ±standard error (SE) was calculated. Student’s *t* tests were performed and *P-*values < 0.05 were considered significant. Mean value of senescence percentages of all experiments ±SE was plotted. Student’s *t* tests were performed and *P*-values < 0.05 were considered significant. Calculations and plots for IC50 and senescence were performed by R (version 3.3.1).

For GE data, the two-sided Wilcoxon–Whitney test was used to check for significant differences between two distributions. The distributions of PFS and OS were estimated using the Kaplan–Meier method and compared with the log-rank test.

### Code availability statement

All codes used to analyze the data and generate the figures are available upon request.

## Electronic supplementary material

Supplementary material

## Data Availability

Cell line annotations, IC50 values and pre-processed expression data from Iorio et al.^20^ cancer cell line dataset were downloaded from http://www.cancerrxgene.org/download. Reverse Phase Protein Array (RPPA) data generated within the MD Anderson Cell Line Project (MCLP)^21^ were downloaded from http://tcpaportal.org/mclp/#/download (version 1.1). IC50 values of palbociclib in breast cancer cell lines from the study of Finn et al.^19^ were also considered. Expression and clinical information from METABRIC study^22^ were downloaded from http://www.cbioportal.org.^45,46^ NeoPalAna GE data^23^ were downloaded from Gene Expression Omnibus (GSE93204) using GEOquery R Package.^47^GE data of our PDS and PDR models are available from the authors upon request.

## Electronic supplementary material

**Supplementary information** accompanies the paper on the *npj Breast Cancer* website (10.1038/s41523-018-0092-4).
