# Supplementary material for: Cyclin E1 and Rb modulation as common events at time of resistance to palbociclib in hormone receptor-positive breast cancer
Source: NPJ Breast Cancer. 2018 Nov 28;4:38. doi: 10.1038/s41523-018-0092-4 (PMC6261939; doi:10.1038/s41523-018-0092-4)
Supplement: Supplementary file 1 — Supplementary material [file 41523_2018_92_MOESM1_ESM.pdf]

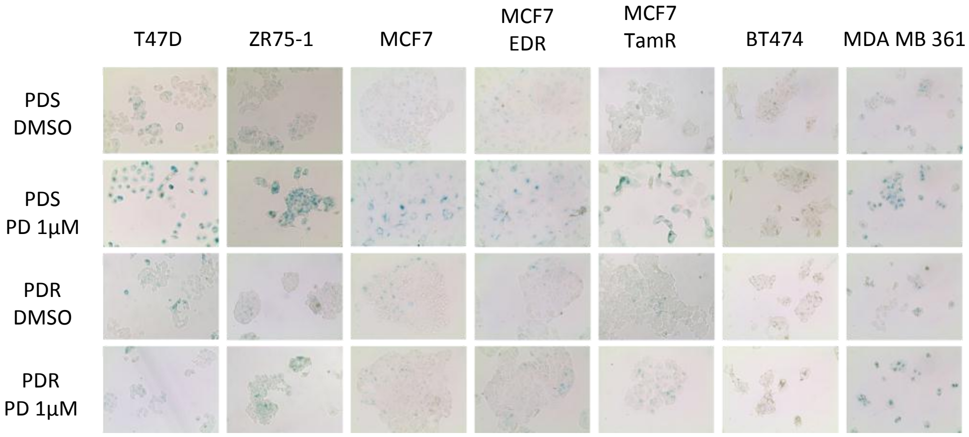

Supplementary Figure s1. Representative images of β-galactosidase senescence assay.

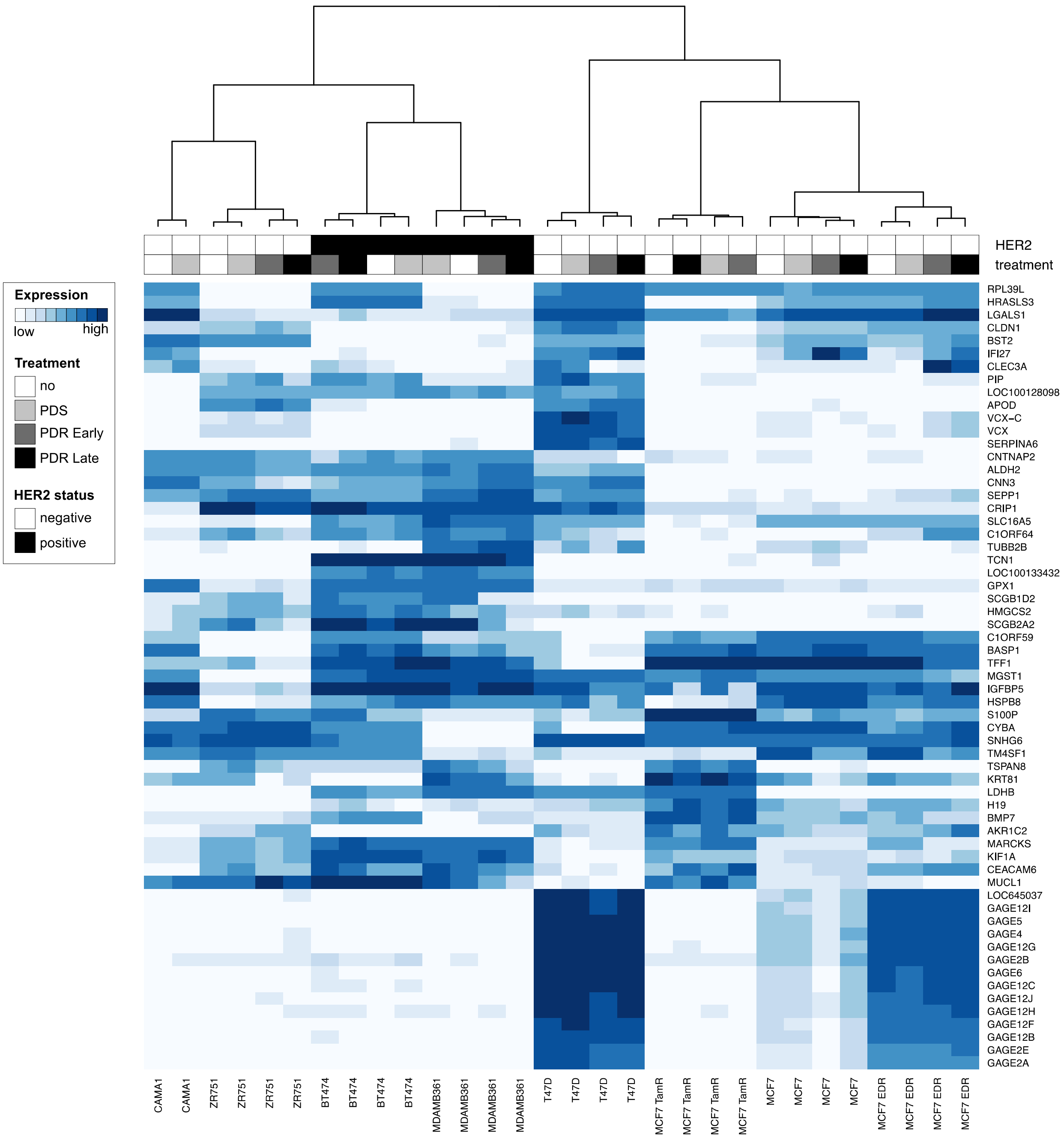

Supplementary Figure s2. Hierarchical clustering of PDS, PDS treated and PDR cell line models (n=30) using ‘1 – Pearson’s correlation’ as distance measure on the expression values of the top 2.5‰ variable genes (n=61). Details about the different treatments and HER2 status are reported in the annotation track above the heat map.

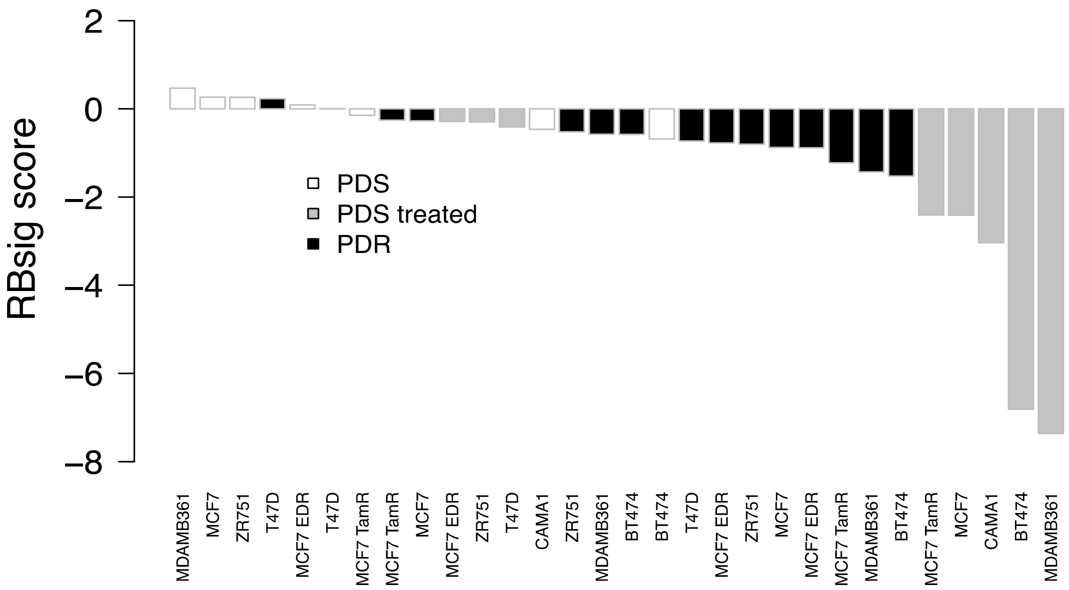

Supplementary Figure s3. Bar plot of RBsig score across PDS, PDS treated and PDR cell line models.

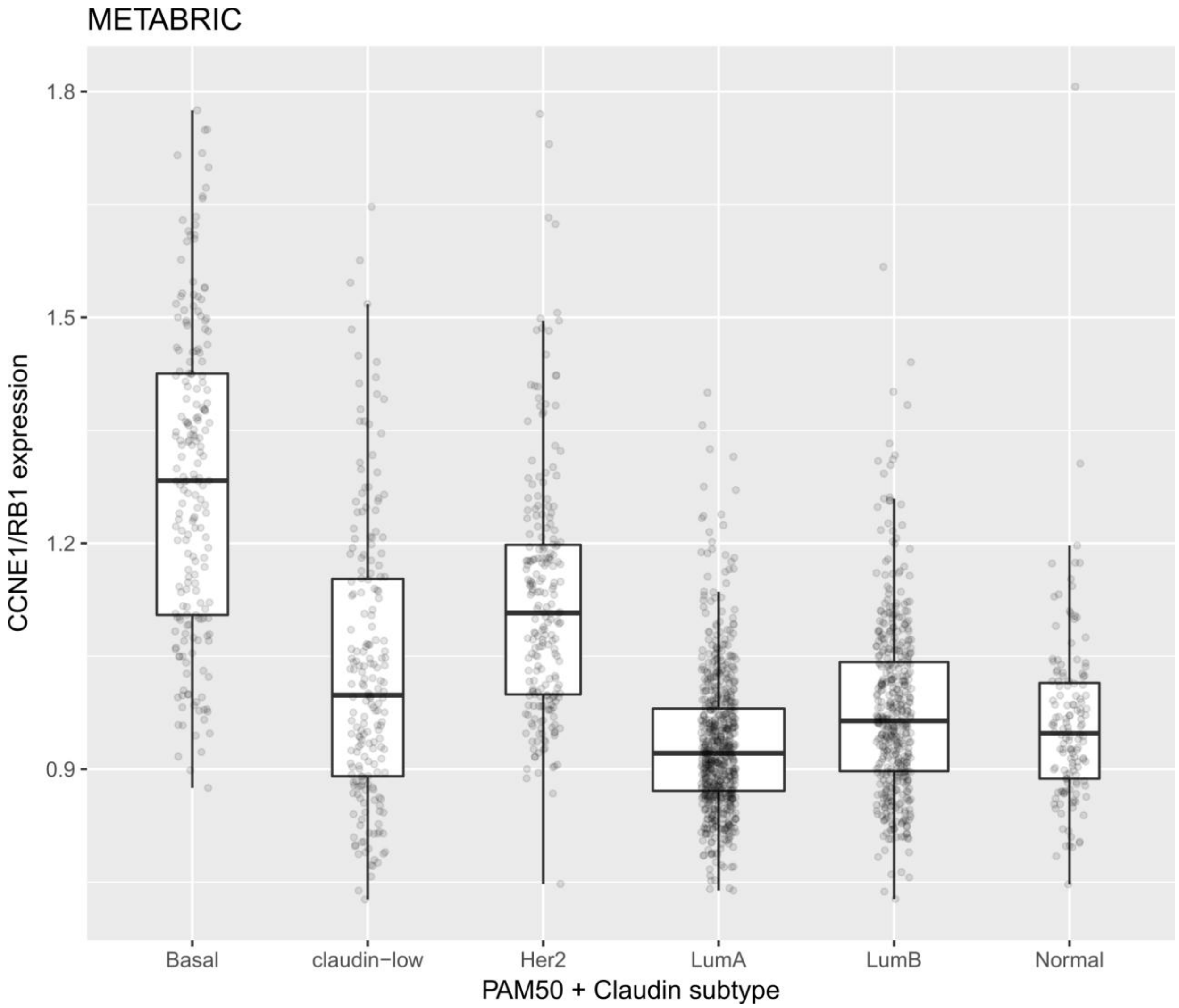

Supplementary Figure s4. Box plots of the distribution of the ratio between the expression values of *CCNE1* to *RB1* across the different PAM50 subtypes in the METABRIC dataset[15].

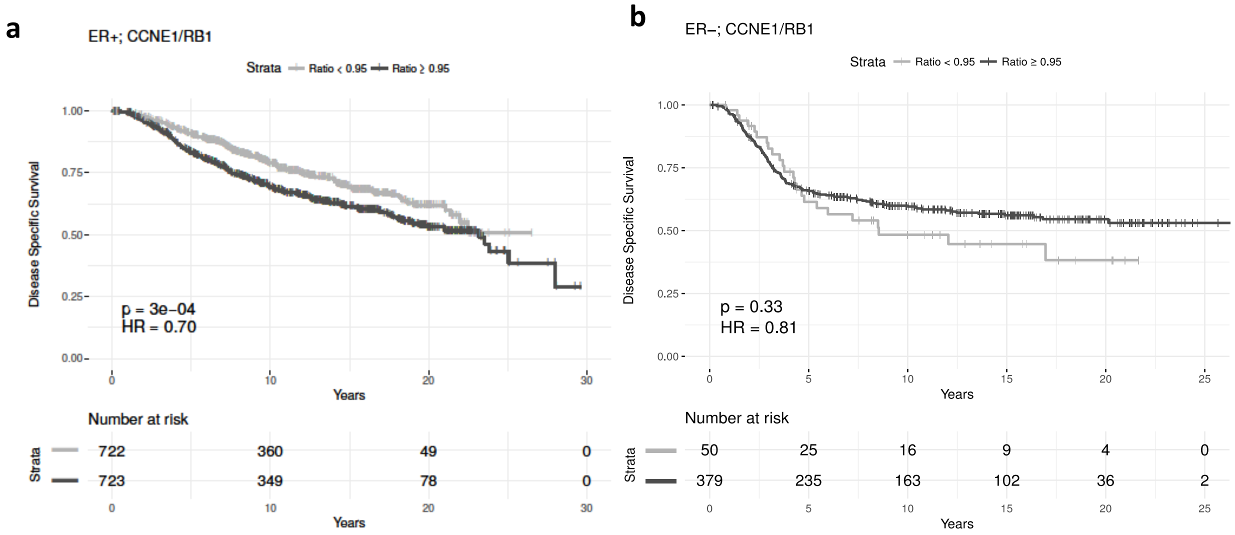

Supplementary Figure s5. a) Kaplan-Meier curves according to high (above the median, dark grey) or low (below the median, light grey) levels of *CCNE1/RB1* in the METABRIC dataset[15] (n=1904) for ER+ (n=1445) samples. b) Kaplan-Meier curves according to high (above the median, dark grey) or low (below the median, light grey) levels of *CCNE1/RB1* in the METABRIC dataset[15] (n=1904) for ER- samples (n=429).

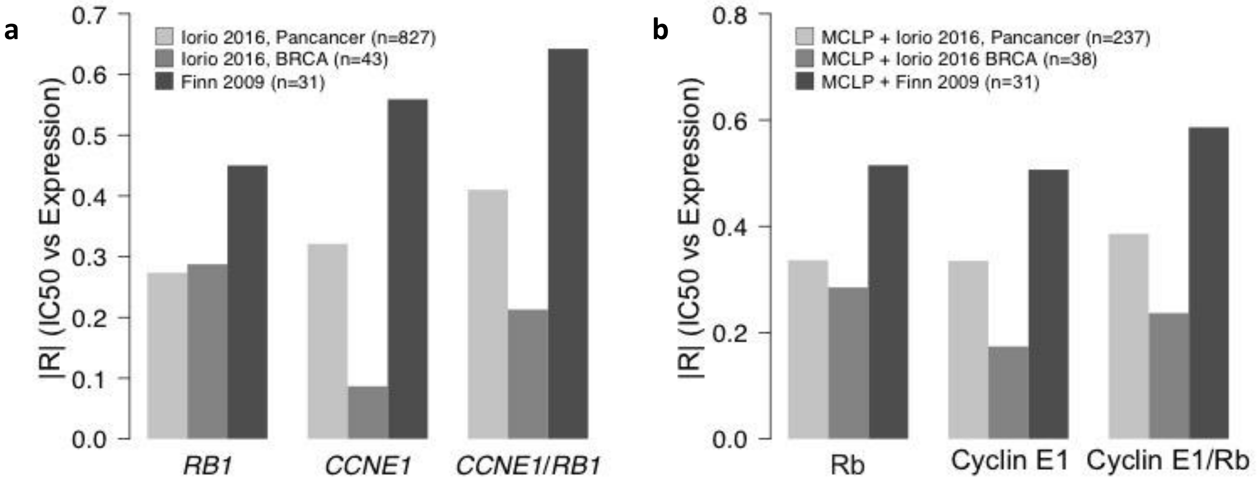

Supplementary Figure s6. a) Bar plot of the absolute value of Pearson's correlation coefficient of IC50 values versus protein expression of *RB1*, *CCNE1* and *CCNE1*/*RB1* by considering both IC50 values and expression data for 827 cell lines of different tumors from Iorio *et al.*, 2016[12] (light grey); both IC50 values and expression data for 43 breast cancer cell lines from Iorio *et al.*, 2016[12] (middle grey); IC50 values and expression data for 31 breast cancer cell lines from Iorio *et al.*, 2016[12] and Finn *et al.*, 2009[14], respectively (dark grey). b) Bar plot of the absolute value of Pearson's correlation coefficient of IC50 values versus expression of Rb, Cyclin E1 and Cyclin E1/Rb by considering IC50 values and protein expression data for 237 cell lines of different tumors from Iorio *et al.*, 2016[12] and MCLP[13], respectively (light grey); IC50 values and protein expression data for 38 breast cancer cell lines from Iorio *et al.*, 2016[12] and MCLP[13], respectively (middle grey); IC50 values and protein expression data for 31 breast cancer cell lines from Finn *et al.*, 2009[14] and MCLP[13], respectively (dark grey).

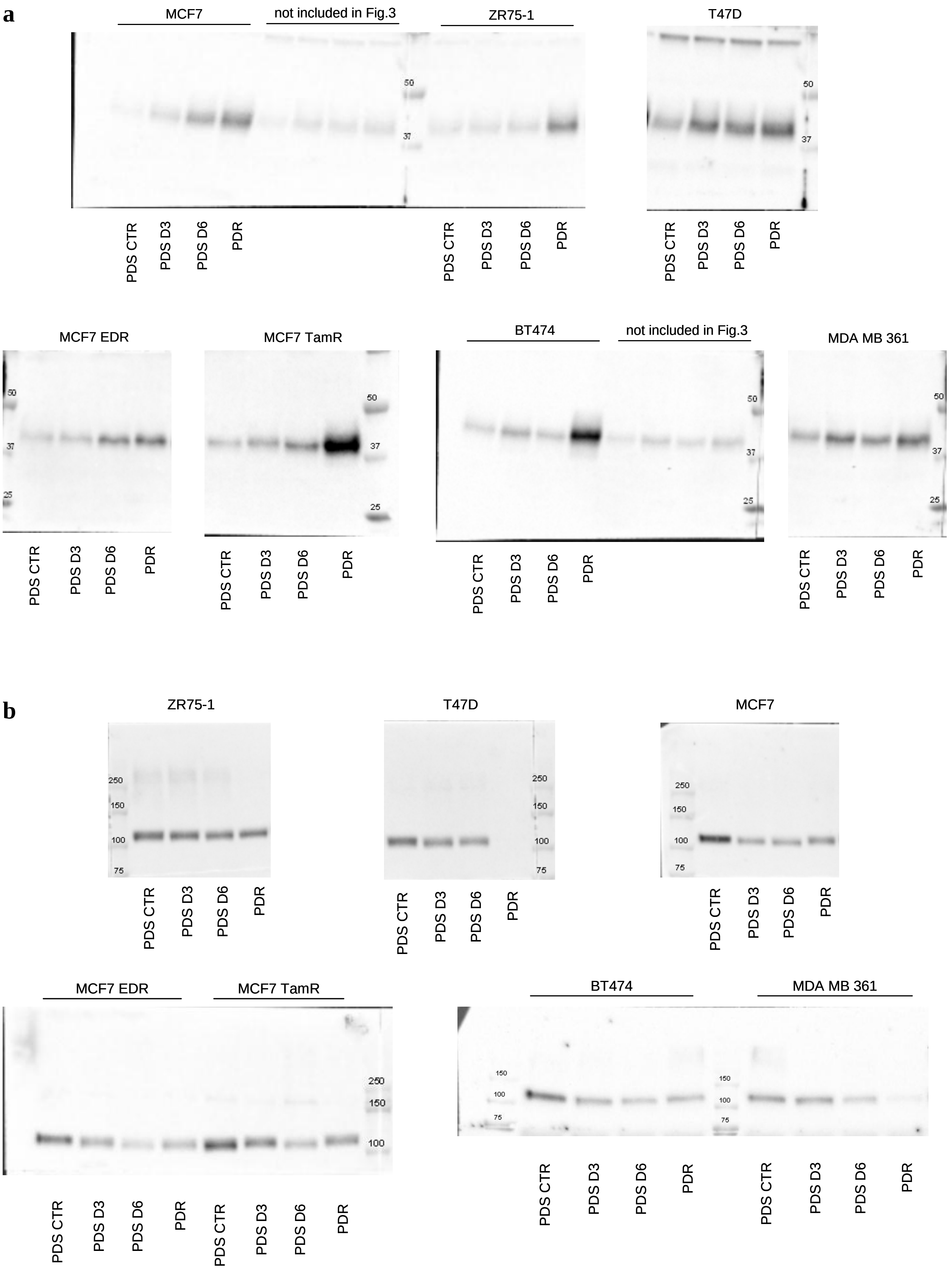

Supplementary Figure s7. Uncropped scans of cyclin E1 (a) and Rb (b) blots

Supplementary Table s1: IC50 values of PDS and PDR models. IC50 values were assessed after 9 days by methylene blue assay. Mean value of IC50 of three independent experiments +/- standard error (SE) was calculated (P-values <0.05, by Student's t test, were considered significant).

	IC50 PDS (SE) [nM]	IC50 PDR (SE) [nM]	IC50 PDR/IC50 PDS	IC50 PDR/IC50 PDS P-value
T47D	180.6 (13.5)	5766.2 (30.6)	31.9	0.000
ZR75-1	78.3 (10.1)	1429.0 (131.1)	18.3	0.009
MCF7	670.2 (79.7)	4112.8 (784.3)	6.1	0.048
MCF7 EDR	18.1 (3.7)	580.4 (102.0)	32.1	0.031
MCF7 TamR	309.5 (44.6)	5001.2 (112.5)	16.2	0.001
BT474	174.7 (26.3)	3073.2 (242.5)	17.6	0.007
MDA MB 361	216.2 (31.2)	2653.2 (293.0)	12.3	0.014
CAMA1	101.0 (28.5)	NA	NA	NA

Supplementary Table s2: Normalized expression (FC, see Methods) of the top 100 differentially expressed genes for PDS treated and PDR cell lines. Columns 2 and 3 report averaged (i.e., by mean) FC values. Differential expression between PDS treated and PDR cell lines is calculated by Wilcoxon-Mann-Whitney test (column 4). Resulting p-values are adjusted by Benjamini Hochberg correction (column 5).

GeneID (HGNC)	PDS treated (FC)	PDR (FC)	P-value PDR vs PDS treated (WMW)	Adj P-value (FDR)
LOC645411	-0,110083652	0,21532616	1,25E-05	0,302242237
FPR1	-0,23060604	0,10827435	7,51E-05	0,90672671
CNTNAP2	-0,19271208	-1,073305596	0,000118835	0,957100416
FOS	0,243863753	-0,597847134	0,000187635	1
CCNE1	-0,206864622	1,073067251	0,000281452	1
SYCE2	-0,439702695	-0,007074492	0,000281452	1
EFNB2	0,216479809	-0,714866509	0,000419051	1
ELF1	0,1996513	-0,303259103	0,000419051	1
HS.335413	0,028845452	0,369625895	0,000600432	1
JAG2	-0,132061152	0,288982933	0,000600432	1
TOB2	0,087238226	0,389363543	0,000600432	1
LOC401074	0,035871249	-0,403548612	0,000850611	1
UTP14C	0,146617934	-0,27379496	0,000850611	1
MRPL37	-0,257050105	-0,094243669	0,001175845	1
MTIF3	0,148457856	-0,179777014	0,001175845	1
MUTED	0,145058856	-0,125525875	0,00161366	1
PAM	0,426600634	-0,152727975	0,00161366	1
SLC44A3	0,354837442	-0,011074872	0,00161366	1
WBP11P1	0,159173042	-0,060948698	0,00161366	1
CD58	0,101009028	-0,34012774	0,00217031	1
DHRS7	0,286372012	-0,06211949	0,00217031	1
GLT25D1	-0,150749421	0,182148104	0,00217031	1
LOC646223	0,02217994	-0,321545645	0,00217031	1
LOC646358	0,086064327	-0,229496171	0,00217031	1
RPAIN	-0,166256313	0,028027778	0,00217031	1
TBL1X	-0,03114284	-0,290368787	0,00217031	1
ZNF274	-0,137263148	0,117572506	0,00217031	1
ATP7B	0,209383044	-0,348126158	0,002895831	1
C1ORF27	0,200062836	-0,085729713	0,002895831	1
COQ10A	-0,155120037	0,278661382	0,002895831	1
N6AMT2	0,145846053	-0,17789196	0,002895831	1
NDRG4	0,068042824	0,324150284	0,002895831	1
DHODH	-0,183774849	0,081362269	0,003713278	1
BAZ2B	0,178258073	-0,065866732	0,003802733	1
C16ORF38	0,290284736	-0,016237359	0,003802733	1
CAP2	0,272511767	-0,222774758	0,003802733	1
CCNA1	0,181125585	-0,198687909	0,003802733	1
CCNDBP1	0,33262544	-0,069143591	0,003802733	1
DHDH	-0,196398612	0,134514237	0,003802733	1
DNAJC12	0,427118157	-0,000914239	0,003802733	1

LOC645018	-0,285789064	0,074474228	0,003802733	1
MAST4	-0,2606596	0,175404124	0,003802733	1
NAT6	-0,156234618	0,101242918	0,003802733	1
NRCAM	0,064529211	-0,461146289	0,003802733	1
CRELD1	0,40867371	0,14679551	0,00495356	1
CXCL12	0,372685386	-0,348401384	0,00495356	1
CXORF26	0,1389814	-0,087579432	0,00495356	1
CYP2D7P1	0,075643719	-0,080779408	0,00495356	1
DSE	0,131450304	-0,273675209	0,00495356	1
DYNLL2	-0,191684851	0,071249835	0,00495356	1
ERRFI1	0,296544557	-0,050562421	0,00495356	1
F8A2	-0,060096157	-0,368636667	0,00495356	1
HMGCS2	0,898669527	-0,200977899	0,00495356	1
IKZF4	-0,043543007	0,185814257	0,00495356	1
LOC100133489	-0,141261656	0,155419833	0,00495356	1
MPPED2	0,126355766	-0,368479988	0,00495356	1
NFKB1	0,074306206	-0,233144083	0,00495356	1
POLR2F	-0,30245563	0,035829904	0,00495356	1
RAB20	0,244550809	-0,091427026	0,00495356	1
RAB7A	0,200049299	-0,006506248	0,00495356	1
RDBP	-0,22057093	0,137403184	0,00495356	1
SAMD4B	-0,063063188	0,137098774	0,00495356	1
UBA6	0,082164351	-0,202367235	0,00495356	1
ARPC4	0,067217198	-0,090379758	0,006367076	1
C6ORF173	-0,760443889	-0,034238186	0,006367076	1
C8ORF51	0,030977767	0,283991824	0,006367076	1
CLDN4	-0,014521548	0,3306664	0,006367076	1
CREB5	0,198764684	-0,118410946	0,006367076	1
DEPDC1B	-0,643640868	-0,189350356	0,006367076	1
GPR98	-0,067179155	-0,398581397	0,006367076	1
LOC100131187	0,203005431	-0,087315038	0,006367076	1
LOC100134370	0,274472143	-0,24252304	0,006367076	1
LOC731049	-0,905534399	-0,14957954	0,006367076	1
MAPK12	-0,124432332	0,306733884	0,006367076	1
POLR2H	-0,173800303	0,01368544	0,006367076	1
RAD54B	-0,463291261	-0,03537668	0,006367076	1
RARRES3	-0,094438764	0,558895626	0,006367076	1
RDM1	-0,058209154	0,158044825	0,006367076	1
RPS6KA2	0,384357211	-0,024420578	0,006367076	1
SETBP1	0,053243649	-0,257669907	0,006367076	1
SPOCD1	0,38903287	-0,048784508	0,006367076	1
STAT6	0,261560997	0,071226029	0,006367076	1
TTK	-0,837477834	-0,075720263	0,006367076	1
VARS	0,147704938	-0,042794633	0,006367076	1
ZNF789	-0,075540639	0,20506801	0,006367076	1
ASAH1	0,105646451	-0,083133756	0,00812459	1
BMI1	-0,087853771	-0,360512386	0,00812459	1
C12ORF24	-0,179408237	0,232263162	0,00812459	1

C6ORF1	0,179589247	0,040074171	0,00812459	1
CCNL1	0,153333764	-0,089016073	0,00812459	1
CGGBP1	-0,014048652	-0,318930478	0,00812459	1
FAHD2B	0,176107377	0,437570788	0,00812459	1
FAM5C	0,167786417	-0,489243505	0,00812459	1
FMO5	0,349333081	0,013545757	0,00812459	1
HS.127242	-0,019305103	0,170883787	0,00812459	1
HS.290694	-0,086962278	0,136011899	0,00812459	1
HS.541226	-0,317506153	0,076922138	0,00812459	1
ITM2B	0,189482934	-0,191279326	0,00812459	1
LOC100129268	-0,135632017	0,042684121	0,00812459	1

Supplementary Table s3: Quantification of western blot bands for cyclin E1 and Rb normalized against total proteins

	Cyclin E1					Rb			
	CTR	D3	D6	PDR		CTR	D3	D6	PDR
T47D	1,00	1,46	1,62	2,47		1,00	0,69	0,63	0,00
ZR75-1	1,00	1,24	1,37	6,63		1,00	0,88	0,75	0,59
MCF7	1,00	4,91	8,14	16,23		1,00	0,30	0,29	0,36
MCF7 EDR	1,00	1,62	3,55	5,96		1,00	0,40	0,19	0,34
MCF7 TamR	1,00	2,03	5,38	34,87		1,00	0,76	0,25	0,68
BT474	1,00	1,37	1,23	7,83		1,00	0,38	0,26	0,38
MDA MB 361	1,00	1,88	1,74	3,90		1,00	0,90	0,36	0,13

Supplementary Table s4: Primary and secondary antibodies working conditions.

Antibody	Clone	Company	Dilution	Secondary Antibody
CDK2	78B2	Cell Signaling Technology	1/500 o.n.	anti-R 1/2000 1h
CDK4	D9G3E	Cell Signaling Technology	1/1000 o.n.	anti-R 1/2000 1h
Cyclin A1	BF683	Merk Millipore	1/1000 o.n.	anti-M 1/2000 1h
Cyclin D1	92G2	Cell Signaling Technology	1/1000 o.n.	anti-R 1/2000 1h
Cyclin E1	HE12	Cell Signaling Technology	1/1000 o.n.	anti-M 1/2000 1h
Cyclin E2		Cell Signaling Technology	1/500 o.n	anti-R 1/2000 1h
E2F1		Cell Signaling Technology	1/1000 o.n.	anti-R 1/2000 1h
E2F2	EPR8622	Abcam	1/2000 o.n.	anti-R 1/2000 1h
GAPDH	D16H11	Cell Signaling Technology	1/10000 1h	anti-R 1/2000 1h
p107	C-18	Santa Cruz Biotechnology	1/1000 1h	anti-R 1/2000 1h
p130	EP2141Y	Abcam	1/1000 1h	anti-R 1/2000 1h
p16	EP435Y-129R	Abcam	1/250 o.n.	anti-R 1/2000 1h
p21	12D1	Cell Signaling Technology	1/1000 o.n.	anti-R 1/2000 1h
p27	D69C12	Cell Signaling Technology	1/1000 o.n.	anti-R 1/2000 1h
pRb S807/811	D20B12	Cell Signaling Technology	1/2000 o.n	anti-R 1/2000 1h
Rb	4H1	Cell Signaling Technology	1/8000 o.n.	anti-M 1/5000 1h
o.n. = 4°C overnight				
1h = 1 hour room temperature				
anti-R = anti-rabbit				
anti-M = anti-mouse				
